# In vivo targeted molecular imaging for activated platelets by mri using USPIO-fucoidan in rat abdominal aortic aneuryms model

**DOI:** 10.1186/1532-429X-13-S1-P372

**Published:** 2011-02-02

**Authors:** Michimasa Suzuki, Jean-Michel Serfaty, Laure Bachelet, Anne Beilvert, Liliane Louedec, Frederic Chaubet, Jean-Baptiste Michel, Didier Letourneur

**Affiliations:** 1 INSERM U698, Paris, France

## Background

P-selectin, expressed on the activated platelets and endothelial cells, is one of the key adhesion molecules that regulate leukocyte trafficking in atherosclerosis. The natural and canonical ligand of selectins is a specific tetrasaccharide structure known as 6-sulfo sialyl Lewis X (SLeX). Fucoidans refer to a type of polysaccharide that contains L-fucose and sulfate ester groups, mainly derived from brown seaweed, and binds strongly to P-selectin. We assessed the hypothesis that a new MRI contrast agent made of fucoidan conjugated with ultra small paramagnetic iron oxide (USPIO) can demonstrate the presence of activated platelets in thrombus of an expanding aneurysm.

## Methods & Results

Thirty Wistar rats were locally infused with elastase. Six days after surgery, rats were set on a 4.7T MRI system and scanned before and after the administration of contrast agent. We used a T2* fast spoiled gradient echo sequence (TR/TE=545.6/6.6msec: FA, 60°: ETL=1: 1mm slice thickness: matrix=384x384: FOV=720x330mm) to image the aorta. Images were acquired every 15 minutes during 1 hour. In a first group, 16 rats were injected with USPIO-CMD-Fucoidan ; in a second group, 4 rats were injected with USPIO-carboxyl methyl dextran (CMD), which was used as a non-specific contrast agent;in a third group, 3 rats received Ferumoxtran-10. Aortic wall signal intensity was measured on MRI slices showing the aortic aneurysm and normalized to the signal intensity of the lumbar muscle of the same section. The percentage of normalized signal intensity enhancement (NSE%) was measured using the following equation: NSE%=(Wi POST-Wi PRE)/Wi PREX100. Comparison between MRI and histology was performed using Alcian Blue, Perls Blue and P-selectine stains. Twenty-three rats developed an aneurysmal dilatation, 7 rats died before MRI. There was no difference in aneurysmal development between the 3 groups (mean diameter, 5.20±2.1mm). We found a negative NSE in all aneurysms of group 1 beginning 30 minutes after injection of contrast media (mean NSE =-21.9±9.4, 1 hour after injection), but no negative NSE in group 2 and 3 rats (mean NSE = 2.7±6.5, 8.9±6.5 1 hour after injection). Correlation between MRI and histology showed an accurate correspondencae between areas of negative NSE and thrombus and P-selectin location.

## Conclusion

We present a new contrast agent based on USPIO and fucoidan, with capabilities to depict quickly and specifically activated platelets. Such contrast agent might be useful to non-invasively detect thrombus.

